# Maximum-likelihood estimation of glandular fraction for mammography and its effect on microcalcification detection

**DOI:** 10.1007/s13246-025-01540-2

**Published:** 2025-05-06

**Authors:** Bryce J. Smith, Joyoni Dey, Lacey Medlock, David Solis, Krystal Kirby

**Affiliations:** 1 Department of Physics and Astronomy, Louisiana State University, Baton Rouge, LA, USA; 2 Physics Department, Mary Bird Perkins Cancer Center, Baton Rouge, LA, USA

**Keywords:** Mammography, Glandular fraction, Monte Carlo, Maximum-likelihood estimation, Effective energy

## Abstract

Breast tissue is mainly a mixture of adipose and fibro-glandular tissue. Cancer risk and risk of undetected breast cancer increases with the amount of glandular tissue in the breast. Therefore, radiologists must report the total volume glandular fraction or a BI-RADS classification in screening and diagnostic mammography. In this work, a Maximum Likelihood algorithm accounting for count statistics and scatter is shown to estimate the pixel-wise glandular fraction from mammographic images. The pixel-wise glandular fraction provides information that helps localize dense tissue. The total volume glandular fraction can be calculated from pixel-wise glandular fraction. The algorithm was implemented for images acquired with an anti-scatter grid, and those without using the anti-scatter grid but followed by software scatter removal. The work also studied if presenting the pixel-wise glandular fraction image alongside the usual mammographic image has the potential to improve the contrast-to-noise ratio on micro-calcifications in the breast. The algorithms are implemented and evaluated with TOPAS Geant4 generated images with known glandular fractions. These images are also taken with and without microcalcifications present to study the effects of glandular fraction estimation on microcalcification detection. The algorithm was then applied to clinical images with and without microcalcifications. For the TOPAS simulated images, the glandular fraction was estimated with a root mean squared error of 6.6% for the with anti-scatter-grid cases and 7.6% for the software scatter removal (no anti-scatter grid) cases for a range of 2–9 cm compressed breast thickness. Average absolute errors were 4.5% and 4.7% for a range of 2–9 cm compressed breast thickness respectively for the anti-scatter grid and software scatter-removal methods. For higher thickness and glandular fraction, the errors were higher. For the extreme case of 9 cm thickness, the glandular fraction estimation yielded 5%, 13% and 16% mean absolute errors for 20%, 30% and 50% glandular fraction. These errors lowered to 1.5%, 9% and 13.2% for a narrower spectrum for the 9 cm. Results from clinical images (where the true glandular fraction is unknown) show that the algorithm gives a glandular fraction within the average range expected from the literature. For microcalcification detection, the contrast-to-noise ratio improved by 17.5–548% in clinical images and 5.1–88% in TOPAS images. A method for accurately estimating the pixel-wise glandular fraction in images, which provides localization information about breast density, was demonstrated. The glandular fraction images also showed an improvement in contrast to noise ratio for detecting microcalcifications, a risk factor in breast cancer.

## Introduction

Breast volume glandular fraction (VGF) is a measure of the extent of glandular tissue present in the breast. It is calculated as the ratio of volume of fibroglandular tissue to total (fibroglandular and adipose) tissue in the breast. Higher breast density is considered a risk factor for cancer [1]. The higher amount of glandular tissue in dense breasts can obscure the visualization of tumors, increasing the likelihood that they remain undetected [2]. Radiologists need an estimated volume glandular fraction (VGF) for screening and diagnostic mammography because mammographic density can vary significantly among patients and change over time [1–3]. To assess breast composition, the American College of Radiology developed the Breast Imaging Reporting and Data System (BI-RADS), which categorizes breast composition into four categories: 1 (predominantly fatty), 2 (scattered fibro-glandular densities), 3 (heterogeneously dense), and 4 (extremely dense). BI-RADS is used to assess breast cancer risk, but its reliability and validity are still debated [4]. A limitation of BI-RADS is that it reports glandular fractions using descriptive text, lacking a quantitative value [5]. Most importantly, the United States FDA has recently standardized the breast density information provided to patients by mammogram facilities. Physicians are required to provide a fibro-glandular density estimate and communicate to patients the impact of breast density on future diagnoses and imaging [6].

Convolutional neural networks (CNNs) have recently been applied to predict breast density, showing comparable performance to human readers [7, 8]. For instance, the results of Deep-LIBRA (Maghsoudhi et al., *Med. Imag. Anal*, 2021) [7] and that of an expert reader were highly correlated (Spearman correlation coefficient 0.9). In a 2022 study by Magni et al. in *Radiology* [8], CNN achieved 89.3% accuracy in distinguishing between BI-RADS non-dense and BI-RADS dense-breast categories. While AI-based methods are efficient and improve hospital workflow and reduce workload of Radiologists, they still rely on the qualitative BI-RADS categorization system, which may introduce a degree of subjectivity.

Physics-based studies have been conducted in the past to get a quantitative value to complement BI-RADS such as van Engeland et al. [9], where relative ratio of glandular tissue and adipose is considered. A calibration with an “almost pure fatty tissue pixel” is needed. Using MRI breast volume as standard, the average error for 22 patients was 13.6%. Heine et al. [10] used breast-tissue equivalent physical phantoms to calibrate the mammograms to reduce inter-acquisition differences such as target/filter, X-ray voltage setting, compressed thickness, etc. In Heine et al. [10], the calibration step takes into account differences in adipose breast tissue and adipose-equivalent tissue phantoms which are often used for calibration. The calibrated percent density (PDc), the calibrated percent glandular fraction (PGc), and the standard percent density (PD), which involves user-operated binary labeling of pixels above a certain attenuation threshold, were all correlated with each other and each predicted cancer, though PGc was less predictive. The methods in [9, 10] are based on raw images. Cheddad et al. [11] performed the area and volume-based density measures on the *processed* for-presentation Full Field Digital Mammography (FFDM) images and compared to Volpara, an FDA approved algorithm [12]. The Volpara algorithm uses acquisition parameters and works on *raw* acquired images but requires a pure fatty region for internal reference [12, 13].

In this study, the glandular fraction (GF) will be estimated as a thickness ratio of the fibro-glandular tissue thickness to the breast tissue thickness, pixel by pixel, from the raw mammography acquired image. The advantage of providing a pixel-wise GF image is that it provides *localization information* of higher density tissue. The corresponding VGF can be derived from pixel-wise GF.

This work is most similar to the work done by Highnam et al. [14], with key differences. The method used in [14] is a mathematical formulation without optimization and has some estimated errors of glandular thickness exceeding 1 cm in the worst cases. In contrast, the current work accounts for Poisson count statistics and scatter, iteratively optimizing the Poisson likelihood function.

Since the large amount of scatter in the mammography energy range tends to degrade image quality, scatter reduction techniques will improve image quality and aid in the estimation of the glandular fraction (GF). The study will consider two different scatter reduction techniques. One is scatter reduction via standard hardware, such as anti-scatter grids (ASG) [15], and second is via software-based scatter correction algorithms. Anti-scatter grids can eliminate up to 85% of incident scatter but have some drawbacks. The ASG absorbs primary X-rays requiring increased exposure to maintain the same fluence at the detector, which results in increased dose to the patient. The use of software-based algorithms has the advantage of no dose increase and no potential artifacts that can arise from a physical grid. One of the leading software for gridless scatter removal in mammography is the proprietary software PRIME, from Siemens Healthcare [16–19]. Various studies have shown that this algorithm successfully removes scatter comparable to an anti-scatter grid, and achieves dose reduction of 13.5–36.4% by eliminating the anti-scatter grid [19]. It is noted that an iterative algorithm for scatter removal in chest radiography also achieve dose reduction while effectively removing scatter comparable to anti-scatter grids [20].

In gridless acquisitions, as with mammography image presentation, scatter correction is essential for accurately estimating the glandular fraction. Our approach integrates scatter estimation and correction directly into the physical model used for glandular fraction estimation.

Microcalcifications represent a known risk factor in breast cancer [21]. Therefore, a study was also undertaken to investigate whether the estimated pixel-wise GF image could enhance the clinical detection of microcalcifications, particularly in challenging cases.

In what follows is a two-step algorithm that was developed first to remove scatter and then estimate glandular fraction: (1) a maximum log-likelihood algorithm based on Poisson statistics to remove scatter algorithmically (ML-ScRmv) or via an equivalent of an anti-scatter grid (ASG-ScRmv) and then (2) estimate the glandular fraction (ML-GF). The poly-energetic spectrum was approximated by an equivalent mono-energetic spectrum using the concept of effective attenuation [22, 23]. The algorithm was tested with TOPAS Monte Carlo generated images. Microcalcification contrast to noise ratio was also assessed in the GF image versus the original mammography image. Finally, the algorithm was applied to different views of clinical images from a patient for pixel-wise GF estimation and microcalcification contrast-to-noise assessment for preliminary clinical testing.

## Methods

**Fig. 1:**
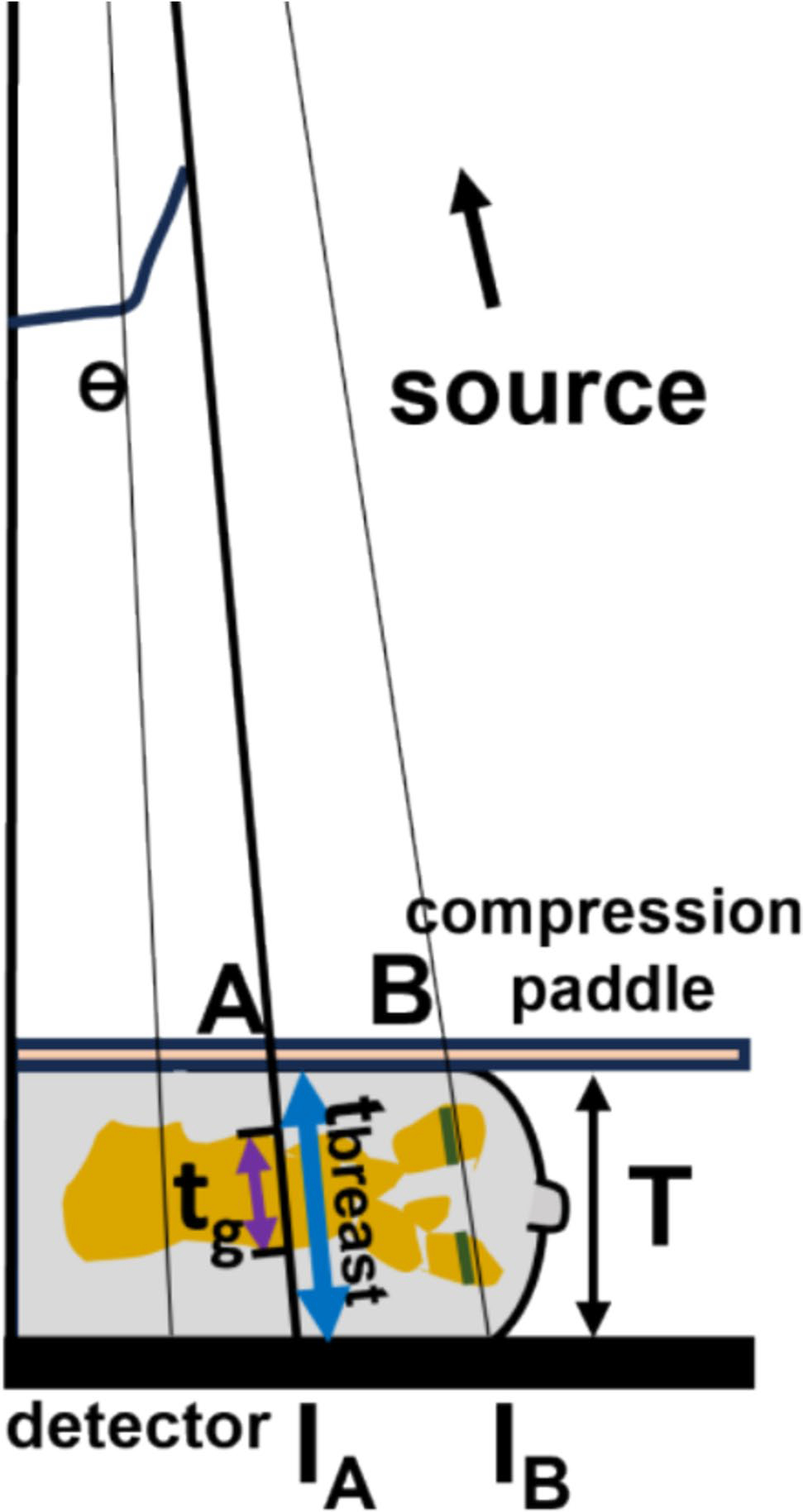
Schematic for ray-by-ray (detector pixel to source) fibro-glandular tissue estimation. The diagram is to relative scale of a 6 cm thickness breast, and source-to-detector distance of 70 cm. The figure is truncated due to high aspect-ratio. **T** is the compressed thickness. Rays A and B travel through skin, adipose and fibrolandular tissue. The slanted thickness **t**_**breast**_ (blue) is the intercept of Ray A through the entire breast and purple (t_g_), and green sections are the length(s) through the glandular part for A and B. The Glandular Fraction GF is **t**_**g**_/**t**_**breast**_ which we wish to estimate for each detector pixel

Not considering scatter for the moment and focusing on rays that traverse entirely through breast tissue after leaving the paddle (e.g., Rays A and B in Fig. 1) before reaching the detector, the net attenuation coefficient μ at a pixel can be estimated using the equation $I_{m}=I_{0}\exp(-\mu L)$, provided the total traversal length *L* (or *t*_*total*_) through paddle and breast is known. Using a three-tissue model (skin, adipose, fibroglandular) of the breast, the net attenuation coefficient μ comprises of the attenuation from the paddle and these three breast tissue components, each weighted by their relative lengths with respect to *L*. By approximating or knowing the thickness of the paddle, skin, and compressed breast tissue, and using known attenuation values for the paddle and the three tissues, the only remaining unknown—the fraction of glandular tissue relative to *L*—can be determined. This estimate can then be further refined by excluding the paddle, to represent the true glandular fraction, defined as the glandular tissue length t_g__ as a fraction of the total intercept of the ray through the breast t_breast_ at each detector pixel.

Several factors complicate this otherwise straightforward analysis. First, the measurement $I_{m}$ is noisy and can be approximately modeled as Poisson-distributed. Second, the X-rays are Compton scattered introducing deviations from their nominal paths. While an anti-scatter grid (ASG) is typically employed to mitigate effects scatter, in some cases, scatter is removed algorithmically as mentioned in the introduction [16–20]. Effects of the polychromatic spectrum on attenuation values and scatter has to be taken into account as well. Another issue is that the rays at the margins, such as those beyond Ray B, encounter the curved part of the breast, leading to traversal through air gaps. In the literature, this marginal region is accounted for using thickness correction methods [24–27], which are fairly standard [9]. In the following analysis, both Poisson statistics and scatter was modeled, the latter important in scenarios where an anti-scatter grid is not used. The polychromatic spectrum was approximated by effective energy. However, within the scope of this paper, we focus exclusively on rays that traverse entirely through tissue without encountering air gaps (e.g., Rays A and B). It should be noted that even for rays beyond Ray B, the proposed method remains valid after applying thickness equalization techniques, such as those employed by Engeland et al. [27].

### Iterative Maximum Likelihood (ML) algorithms to estimate GF

The method involves two steps. In the first step, given a raw mammography image, a scatter correction is applied via an iterative ML algorithm. This step is not required if an anti-scatter grid was present during acquisition. In the second step, the GF is estimated by employing a second iterative ML algorithm.

#### Step 1. ML for scatter correction. (*ML-ScRmv*)

Similar to the maximum likelihood method implemented to estimate thickness in previous literature [28], here μ, the linear attenuation coefficient, is estimated assuming that the compressed breast thickness is known from the paddle information in the DICOM header of the clinical FFDMs.

The scatter was modeled by the following two equations, where f(μ) is the likelihood function assuming that the measured counts $I_{m}$ with object in place is Poisson distributed. For a single energy,

(1) $$f(\mu )=\left(I_{m}\right)\;ln\left(I_{e}\right)-I_{e}$$

The term $I_{e}$ is the expected value of the count, for a given μ and $I_{0}$.

(2) $$I_{c}=I_{0}e^{-\mu t}+Sc.$$

where $I_{0}$ is the counts without object in place, Sc is the modeled scatter and t is the total thickness of the object traversed by the X-ray, where the total path length takes into account the adipose tissue, glandular tissue, skin, and Lexan (compression paddles). Note both μ and *Sc* are functions of energy. Sc is also a function of thickness t.

Considering Eqs. 1 and 2, the μ was updated iteratively through applying Newton Raphson’s root finding of the first derivative, f′(μ), to maximize f(μ) shown in (3) below

(3) $$\mu^{n+1}=\mu^{n}-\frac{f^{\prime }(\mu )}{f^{\prime \prime }(\mu )}$$

where $f^{\prime }(\mu )$ and $f^{\prime \prime }(\mu )$ are the first and second derivatives of (1) with respect to μ. This gave us an updated scatter- corrected count image $I_{q}$ as shown in (4). This was used as input in next step.

(4) $$I_{q}=I_{0}e^{-\mu t}$$

#### Step 2. ML for GF estimation (*ML-GF*)

In Step 2, a second ML algorithm was used to calculate the GFs. This optimization iteratively maximizes the Poisson log-likelihood of X-ray counts without scatter $I_{q}$ as shown in Eq. 4 using Newton Raphson method. This time, the log likelihood equation is written with the scatter-corrected image $I_{q}$. The GF of the phantom is estimated at each detector pixel encountered by the X-ray by using the following Poisson log-likelihood function

(5) $$g\left(m_{1}\right)=\left(I_{q}\right)\;ln\left(I_{e1}\right)-I_{e1}$$

where the expected model was updated to $I_{e1}=I_{0}e^{-\mu_{1}t}$ as the scatter-corrected image.

The total model $\mu_{1}$ is split into four variables to account for the different materials that the X-rays traverse through: paddle material (Lexan) $\left(\mu_{l}\right)$, skin $\left(\mu_{s}\right)$, adipose tissue $\left(\mu_{a}\right)$, and glandular tissue $\left(\mu_{g}\right)$. This leads to the following equation

(6) $$\mu_{1}=\mu_{l}\times j+\mu_{s}\times k+\mu_{a}\times \left(1-k-m_{1}-j\right)+\mu_{g}\times m_{1}$$

It is important to note that $I_{q}$ accounts for attenuation through the breast and paddle. The $m_{1}$ is the ratio of the glandular tissue thickness to the total path length (*paddle and breast*). Similarly, the fraction j is that of paddle (of material Lexan) in path of X-rays, and k is the fraction of skin in path of X-rays. For all these fractions the thickness of each component is divided by $t_{total}$ (*or* L), the total intercept of the ray through the paddle and the breast. The lengths through Lexan and skin, j and k, are assumed known, or estimated. In this work, a Lexan paddle with a thickness of 2.5 mm [29], and a skin thickness of 1.45 mm were assumed [3]. The $m_{1}$ is estimated as follows in Eq. 7 and then corrected by a scale factor to obtain the correct factor m, with respect to $t_{breast}$ as shown in Eq. 8.

The glandular ratio, $m_{1}$, is estimated via the Newton Raphson updates as follows

(7) $$m_{1}^{n+1}=m_{1}^{n}-\frac{g^{\prime }\left(m_{1}\right)}{g^{\prime \prime }\left(m_{1}\right)}$$

where $g\prime \left(m_{1}\right)$ and $g^{\prime \prime }\left(m_{1}\right)$ are the first and second derivatives of (5) with respect to $m_{1}$. To eliminate the compression paddles from the fraction to leave just the length through breast, the glandular fraction $m_{1}$ is first multiplied by the total setup thickness (paddle thickness plus breast thickness), $t_{\mathrm{total}}$ to obtain the net glandular thickness distributed along the ray. Dividing this by the thickness through the breast $t_{\mathrm{breast}}$ yields the final glandular fraction, m.

(8) $$m=\frac{m_{1}\;\times \;t_{\mathrm{total}}}{t_{\mathrm{breast}}}$$

This removes the effect of paddle thickness from the GF-estimate. This method is applied to finding the glandular fraction GF on compressed phantoms made up of primarily glandular and adipose tissue and clinical datasets.

Calculation of volume glandular fraction (VGF) (or volumetric breast density [30, 31]) can be done by finding the lengths of fibro-glandular tissue for each pixel $m\times t_{\mathrm{breast}}$ and summing over all pixels and then dividing the lengths $t_{\mathrm{breast}}$ summed over all pixels.

The algorithm was evaluated using TOPAS, which wraps and extends the GEANT4 Monte Carlo program [32, 33]. TOPAS was used to generate images of a semicylindrical object resembling a breast phantom with a heterogenous mixture of glandular and adipose tissue. In the simulated digital breast phantoms, the scatter-to-primary ratio (SPR) was extracted from individual simulations by separating scatter from primaries post-simulation. This allowed the creation of a pixel-by-pixel SPR map for use to model scatter, Sc. Anti-scatter-grid case was indirectly simulated. The simulations are explained in the next two sections.

### Evaluation with TOPAS monte carlo simulations

TOPAS Monte Carlo (version 3.8.1) software [32, 33] was used to generate realistic X-ray images for a mammography system designed to image compressed breast tissue with a range of thicknesses. The standard breast compressed thickness prescribed by the Mammography Quality Standards Act (MQSA) is 4.2 cm (no. uu [34]). However, there is a wide variation around this value, ranging from about 2 cm to slightly over 8 cm [35–37]. In this work, compressed breast thicknesses of 2 cm–9 cm in steps of 1 cm was simulated. While 20%, 30% and 50% fibroglandular tissue were simulated for all the thicknesses considered, in reality the fibroglandular fraction drops off sharply with thickness of breast [36, 37]. The most common glandular fraction is around 30% (histogram in Fig. 2 in Giese et al. [37]). As observed from the plot in Fig. 1 in Dance et al. [36], a breast with 9 cm compressed and 50% glandular fraction would be extremely rare.

#### TOPAS set-up

**Fig. 2:**
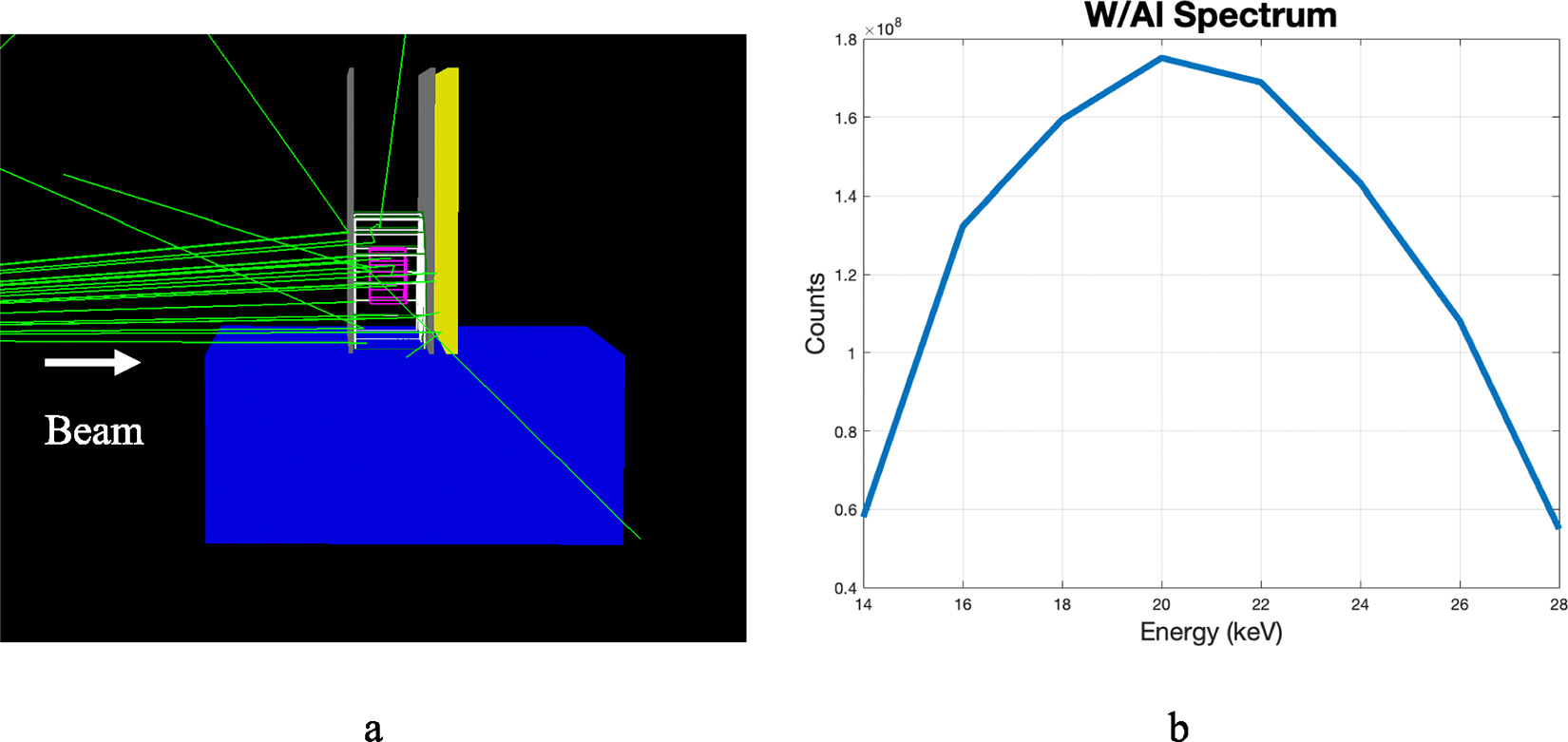
**a** Mammography setup in TOPAS with beam on. Objects from left to right are water bath to simulate body (blue), upstream compression paddle (gray), skin layer (green), adipose tissue (white), glandular tissue (pink), downstream compression paddle (gray), and detector (yellow). **b** W/Al spectrum used to model the energy of the beam [15]

Option4 of the physics list was used, which included photoelectric effect, coherent and incoherent scattering. The detector is a Cesium Iodide detector with dimensions 20*cm* × 15*cm* × 6*mm*. The source to detector distance is 70 cm and the source to distal side of object distance is 68.5 cm. This corresponds to a 1.5 cm air gap, which is a similar setup described in Boone et al. [38]. The source was a 28 kV poly-energetic W/Al (tungsten anode with 0.7 mm aluminum filter) spectrum [15]. The spectrum energy domain was considered from 14–28 keV, peaking around 20 keV. Figure 2 shows the setup with the X-rays turned on. The white wireframe object represents adipose tissue while the pink wireframe object represents glandular tissue. The μ_a_ values were calculated from NIST XCOM database after constructing the mixture based on the materials listed in the GEANT4 materials database for adipose tissue. For glandular tissue, the μ_g_ is from NIST XCOM after constructing the mixture based off the materials listed by Hammerstein et al. [39]. Additionally, the Lexan compression paddles (2.5 mm thick [29]), a 1.45 mm skin layer, and a water bath were included to simulate the body. For each energy bin, 125 million counts were simulated, ensuring sufficient statistical accuracy [40]. The scintillation light or readout was not explicitly modeled. The X-ray interactions are read out directly. For the GF estimation purposes, the detector was binned to 1 mm.

To evaluate the algorithm, breast thicknesses were considered in 1 cm increments from 2 to 9 cm, along with an extreme case of 9 cm, all with a diameter of 15 cm and chest wall to nipple distance of 7 cm [41], and glandular fractions of 20%, 30%, and 50%. The glandular tissue was concentrated within a smaller half-cylindrical volume (pink volume in Fig. 2), surrounded by the larger half-cylindrical adipose volume (as shown in Fig. 2). These numbers were chosen to simulate the average range of glandular breast tissue [2, 3].

**Fig. 3:**
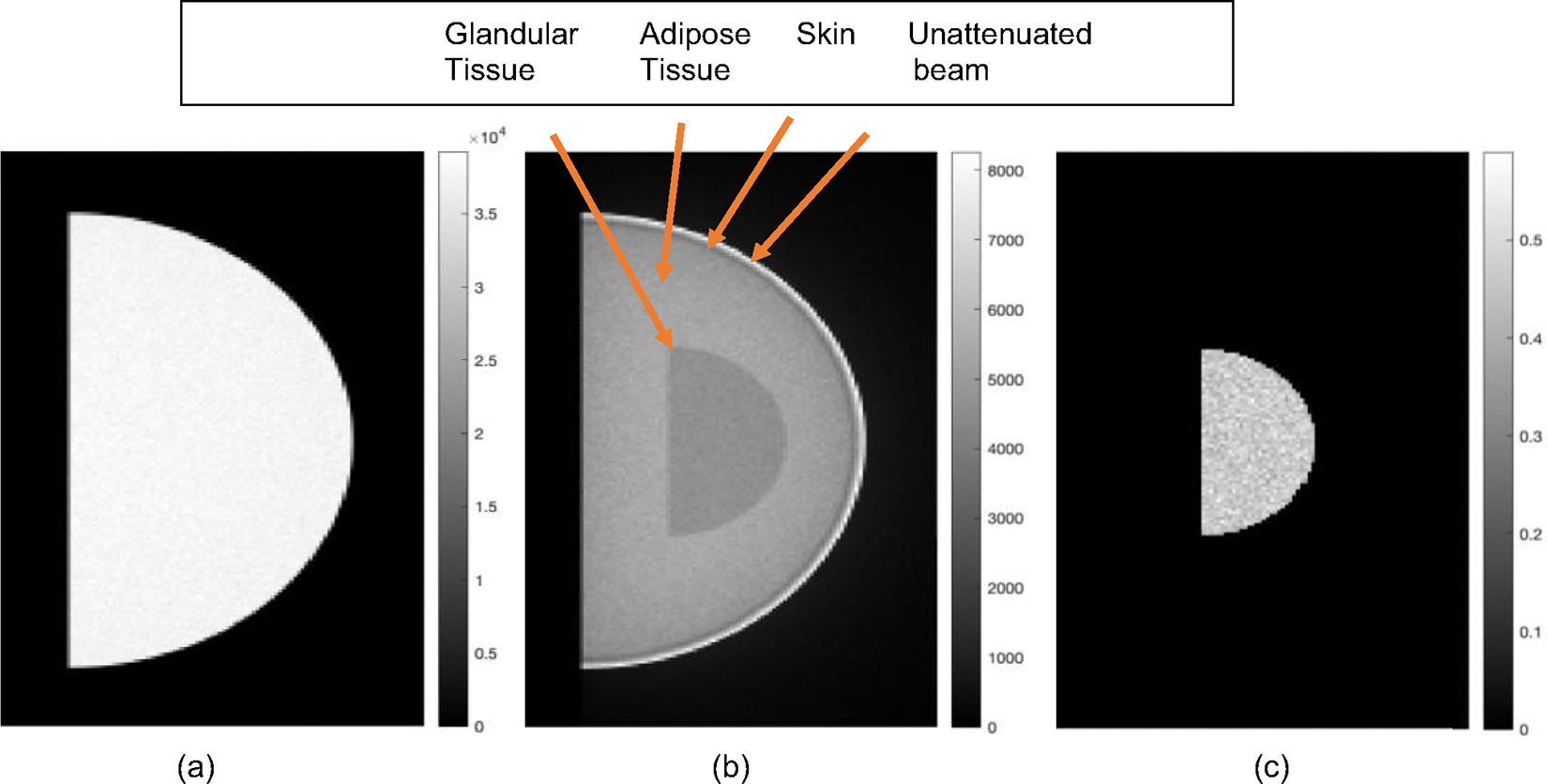
Image obtained from TOPAS **a** $I_{0}$, without object. A semi-circular mask is applied to show only the region of interest. **b** $I_{m}$, with object. A slight portion of the unattenuated beam is also shown. (**c**) is the final GF output image of ML algorithm after steps 1–2 is completed. The color bar in (**a**) and (**b**) show raw counts while that in (**c**) are fractions, representing the glandular fraction

Figure 3a, b show the input $I_{0}$ and $I_{m}$ images used in the ML algorithm to estimate the glandular fraction for an example case, while Fig. 3c shows the outcome of the ML algorithm for this example.

#### Anti-scatter grid scatter removal simulations in TOPAS (*ASG-ScRmv*)

The GF estimation will be evaluated for cases where the software-based scatter correction Eqs. 1–3 is applied as well as for cases where an anti-scatter-grid (ASG) is used instead when acquiring data. For the anti-scatter grid simulation, instead of a direct grid, a flexible method was implemented in TOPAS to achieve the effects of clinical mammography anti-scatter grid. The flexible method could be potentially used to test effects of different types of anti-scatter grid by controlling the levels of remnant scatter. The method is as follows.

**Fig. 4:**
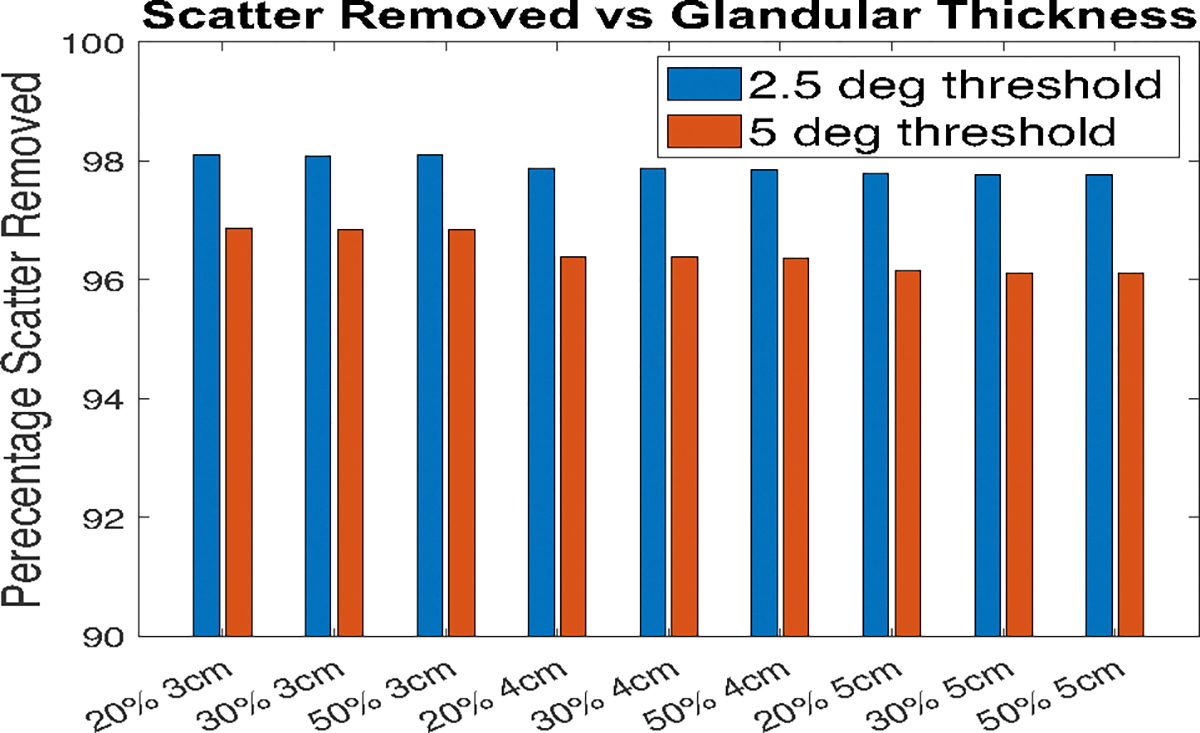
Percentage scatter removed for each setup for 2.5° & 5°

From the TOPAS histories, the direction cosines of the momentum vector with respect to the detector co-ordinates x and y are known for each individual history counted by the detector. Using these x and y components of momentum vectors, the angle that each ray hit the detector are calculated. Any angle larger than a certain value can be estimated to be scatter and removed from the image. Previous literature shows the mean scatter angle for mammography projections is 5–20 °C [42]. Two threshold angles 2.5 °C and 5 °C were tested in this work and the scatter removal thereof was estimated. This was estimated from the counts in the region outside the object which is scattered photons. The amount of scatter was estimated as follows. The X-rays detected approximately 1 mm outside the radius of the (outer) semi-cylinder object and outside of beam were considered scattered rays. The number of counts detected beyond the object (where only scattered photons will reach) were compared before and after thresholding by angle to determine how much scatter was removed. Figure 4 shows the removal by 2.5 and 5 °C. The threshold of 2.5 °C was chosen since it was estimated that this angle removed about 98% of the scatter compared, as shown in Fig. 4. To simulate real clinical conditions, 25% of the removed scatter was then added back since a typical anti-scatter grid only removes 75%–85% of the scatter [15]. This is done by scaling the scatter-only image by 0.25 and adding back to the primary-only image.

This gave an estimated “scatter corrected” $I_{m}$ akin to what would be obtained with ASG and then used as an input to Eq. 5 for the GF estimate via ML.

#### Polychromatic spectrum

In this work, attenuation coefficients at the effective energy were used and estimated the errors thereof using TOPAS. The total equivalent attenuation is calculated first for the given thickness (of object and paddle) for the detector images (obtained with a polychromatic spectrum). The effective energy for this equivalent total attenuation (μ) is found using linear interpolation among the known spectrum range. The attenuation coefficients of adipose tissue ($\mu_{a}$) and glandular tissue ($\mu_{g}$) were estimated for the algorithm at this effective energy, which for a 28 kV W/Al spectrum, ranged from 20.65 to 22.25 keV. This method is then evaluated.

The use of polychromatic versus effective energy method was evaluated in TOPAS. The total X-ray intensity absorbed or scattered was evaluated as $I_{\mathrm{loss}}=1-\frac{I_{m}}{I_{0}}$ for each of poly and effective monochromatic energy cases and then the percent error was calculated as shown below in Eq. 9.

(9) $$\%\;\mathrm{error}=\frac{I_{\mathrm{loss}(\mathrm{poly})}\;-\;I_{\mathrm{loss}(\mathrm{mono})}}{I_{\mathrm{loss}(\mathrm{mono})}}\times 100$$

The percent difference in absorption and scatter are shown in the Results sections (Table 1). Other validations such as poly-energetic versus monoenergetic methods for different glandular thicknesses are also shown and discussed in Results section.

#### Variation due to location

The impact of the location of glandular tissue on the ML-GF estimation was investigated by repositioning the tissue within a simulated breast phantom with thickness 4 cm and 20% glandular fraction. The glandular tissue was placed 1 cm anterior to the center, at the center, and 1 cm posterior, away from the center.

### Addition of microcalcification to monte carlo images

Microcalcifications are considered to be robust markers for breast cancer, where 30–50% of non-palpable cancers are detected by microcalcifications revealed during mammography [43]. This makes detecting them early an important feature of mammography. Detection of type II (hydroxyapatite) calcifications is crucial since those are associated with malignant lesions [44].

Microcalcifications were simulated in the simulated digital breast phantom images by inserting calcium deposits in two different spots of the glandular portion of the 3D object before obtaining the projection data. These deposits are cylindrical in shape with a diameter of 1 mm and a height of 0.5 mm [45].

### Clinical images

#### Comparing ML-GF outputs to clinical display images

The ML-GF algorithm was also tested on raw clinical (DICOM format) images from UPMC Breast Tomography and FFDM Collection [46]. These previously had scatter removed, so no scatter correction step was performed. The value of the compressed breast thickness was available in DICOM header. Where the paddle information was absent, the thickness was assumed to be a 2.5 mm paddle. The skin thickness was assumed to be 1.45 mm (as used in the literature) [3]. Effective energy was estimated using spectrum information in DICOM header (W anode with Ag filter 0.05 mm). Where non-breast tissue was present (such as pectoral muscle) they were segmented out before applying the algorithm.

FFDM clinical images are often not available as raw detector counts. Instead, they undergo a series of processing steps to improve contrast for presentation. Initially, the log-ratio, $\ln(I_{0}/I_{m})$ is calculated, followed by the application of various vendor-specific functions. To facilitate the GF algorithm, it is important to have access to the original raw images.

To conduct the micro-calcification analysis as well, raw images are required. Micro-calcifications are added as attenuated counts within the raw data. Afterward, it’s necessary to estimate the vendor-specific transformation for displaying the images realistically for clinicians’ assessment.

Therefore, just the one clinical case that was available where the raw (count) image and the processed display images were both available was considered. Fortunately, this case had 4 different views of the breast, which effectively created different unique pixel-wise glandular fractions due to different view angles of projection. The ML-GF algorithm was applied for those cases using the linear image. This set was used for the micro-calcification analysis as well.

The $I_{0}$ (counts without the object) that is required for the ML-GF algorithm was estimated from a column of pixels outside the object. In the results, the ML-GF (without any processing) was compared with the “for presentation” high contrast images that were provided. Four images were downloaded of the same breast: right craniocaudal (R CC), left craniocaudal (L CC), right mediolateral oblique (R MLO), and left mediolateral oblique (L MLO), and the clinical images were ran through the same process the simulated digital breast phantom images went through, (except for the scatter-to-primary estimate and scatter correction step because the clinical images had scatter previously removed). For all the clinical images, the spectrum was calculated from some of the data available in the DICOM header, the kVP and W anode/Ag filter. The DICOM header data indicated the voltages were between 33 and 35kVP (depending on view), with a W anode and a 0.05 mm Ag filter. The effective energy was estimated using the same method as the simulated digital breast phantom images and by using a similar setup in TOPAS but with the same parameters listed in the DICOM header.

#### Adding microcalcifications to clinical images and comparing to ML-GF

**Fig. 5:**
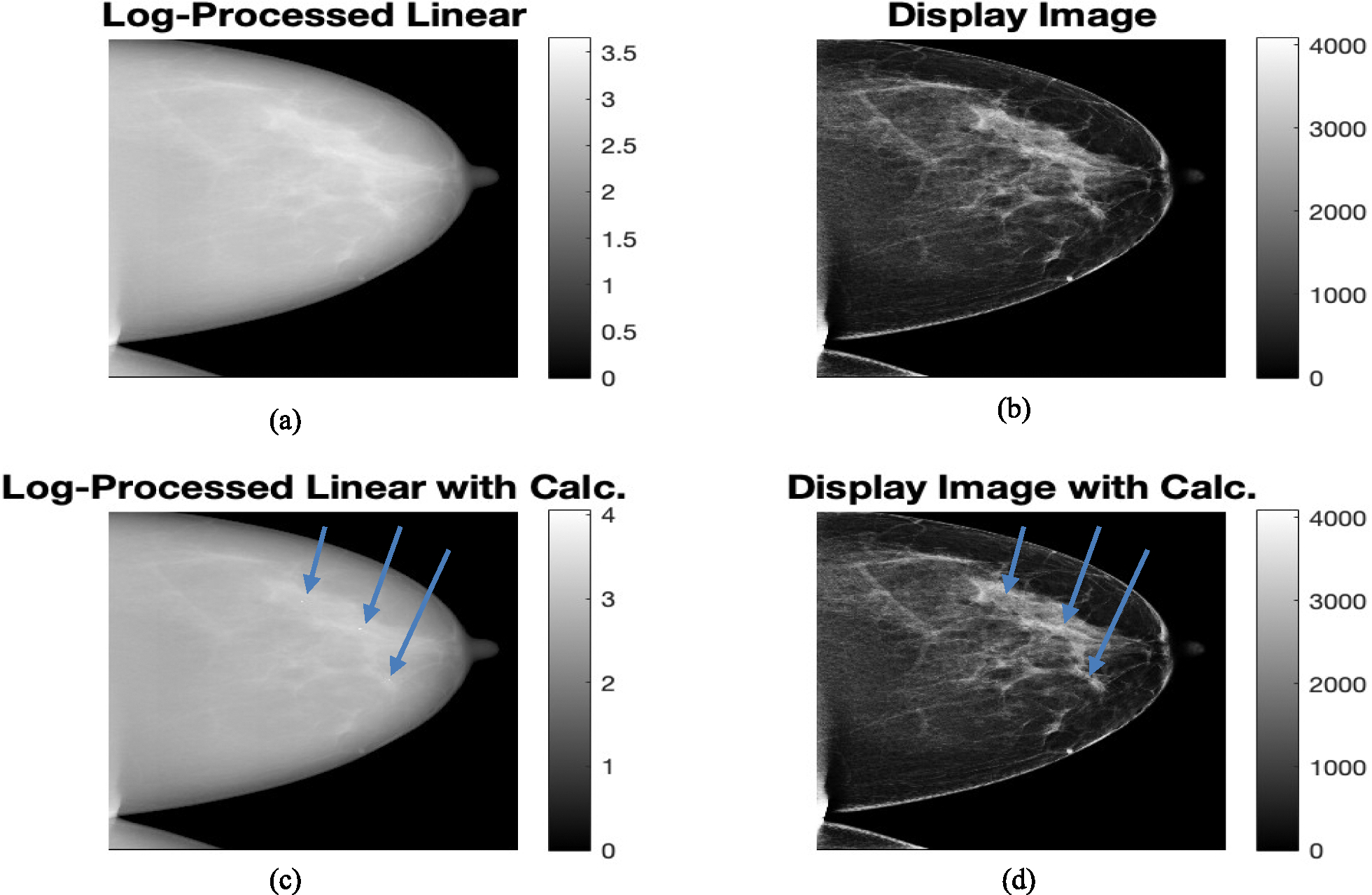
Mapping is obtained from **a** Log-Processed Linear Clinical image to the **b** log-Clinical (Display) image. This mapping is then applied to **c** Log-Processed Linear image with micro-calcifications to obtain **d** Display image with calcification (arrows). The calcification is hardly visible in these images

Microcalcifications were added similar to the simulated digital breast phantom images. These microcalcifications were added as attenuation of raw counts in a denser region of interest in the densest sections of glandular tissue within the actual linear raw clinical images. The attenuation was estimated from the attenuation by micro-calcifications for the simulated digital breast phantoms. This is done at different spots for the four views for the single-patient for whom the raw data is available. Then the ML-GF algorithm was applied. The visibility of microcalcifications on the ML-GF image is to be tested. To ensure a fair display comparison, ML-GF with micro-calcifications is to be compared with a high-contrast clinical image (display version), not raw linear count images. The same processing step that was used in converting raw image to processed image was applied to the raw image added with simulated calcification to generate the corresponding processed images. Details are explained below and shown in Fig. 5.

1. Take the log-ratio $\ln(I_{0}/I_{m})$ of the original raw count images (prior to micro-calcification addition) heretofore called Log-converted or Log-processed “linear” image Fig. 5a.
2. Pixel-wise estimate the vendor mapping between this log-converted images Fig. 5a and the high-contrast clinical display images available for this original set (Fig. 5b)
3. Apply the log-conversion (Fig. 5c) and the estimated mapping in Step 2 above to the log-ratio images with micro-calcifications to obtain display images with calcifications (Fig. 5d)

The calcification is hardly visible in these images. To evaluate any improvement in visibility of the calcifications in these images, these images were compared with the ML-GF images with calcifications by calculating the contrast-to-noise ratio, $CNR=\frac{x_{s}-x_{bg}}{\sigma_{bg}}$,where $x_{s}$ is the signal strength of calcification, $x_{bg}$ is the signal strength of the surrounding glandular tissue and $\sigma_{bg}$ is the standard deviation of the surrounding glandular tissue.

All the code in this work written in Matlab, MathWorks, Natick MA.

## Results

### Topas simulated images

#### Effective energy

Table 1 shows the percent error (as defined in Eq. 9) between the absorption and scatter loss computed at the effective energy and the true measured loss using the polyenergetic W/Al spectrum. The poly-energetic spectrum was a W/Al spectrum. The effective energy values were estimated using linear interpolation of the linear attenuation coefficients. The count loss for each case was calculated as $I_{\mathrm{loss}}=1-\frac{I_{m}}{I_{0}}$ where $I_{m}$ is the total counts across the detector from TOPAS Monte Carlo simulations with object present and $I_{0}$ is the total count without the object. The percent differences in loss between the effective-energy and polyenergetic cases are less than 1% across all glandular fractions (GF) and breast thicknesses. The low error values in Table 1 validate the effectiveness of the approach.

The effective energy for different breast tissue thickness was also tested. The effective energies for the simulated breast phantoms of 2–9 cm thickness ranged from 20.65 keV to 22.25 keV for the 28 kV W/Al spectrum. For the one 9 cm case with W/Ag, the effective energy is 21.40 keV.

In past studies, the SPR was calculated solely as a function of the total breast thickness, however other studies exist showing the SPR could change up to 15% depending on the glandular composition [47]. The SPR was calculated using known scatter from the Monte Carlo histories. Each Monte Carlo simulation was run once with a random seed and included 10^9^ histories. Boone et al. [48] conducted a comprehensive review of SPR in mammography using 10^7^ histories. Given the two orders of magnitude increase in the number of histories in our simulations, we expect the resulting SPR ratios to have low variance, with statistical uncertainties well below 1%.

#### ML-ScRmv/ML-GF and ASG-ScRmv/ML-GF

**Fig. 6:**
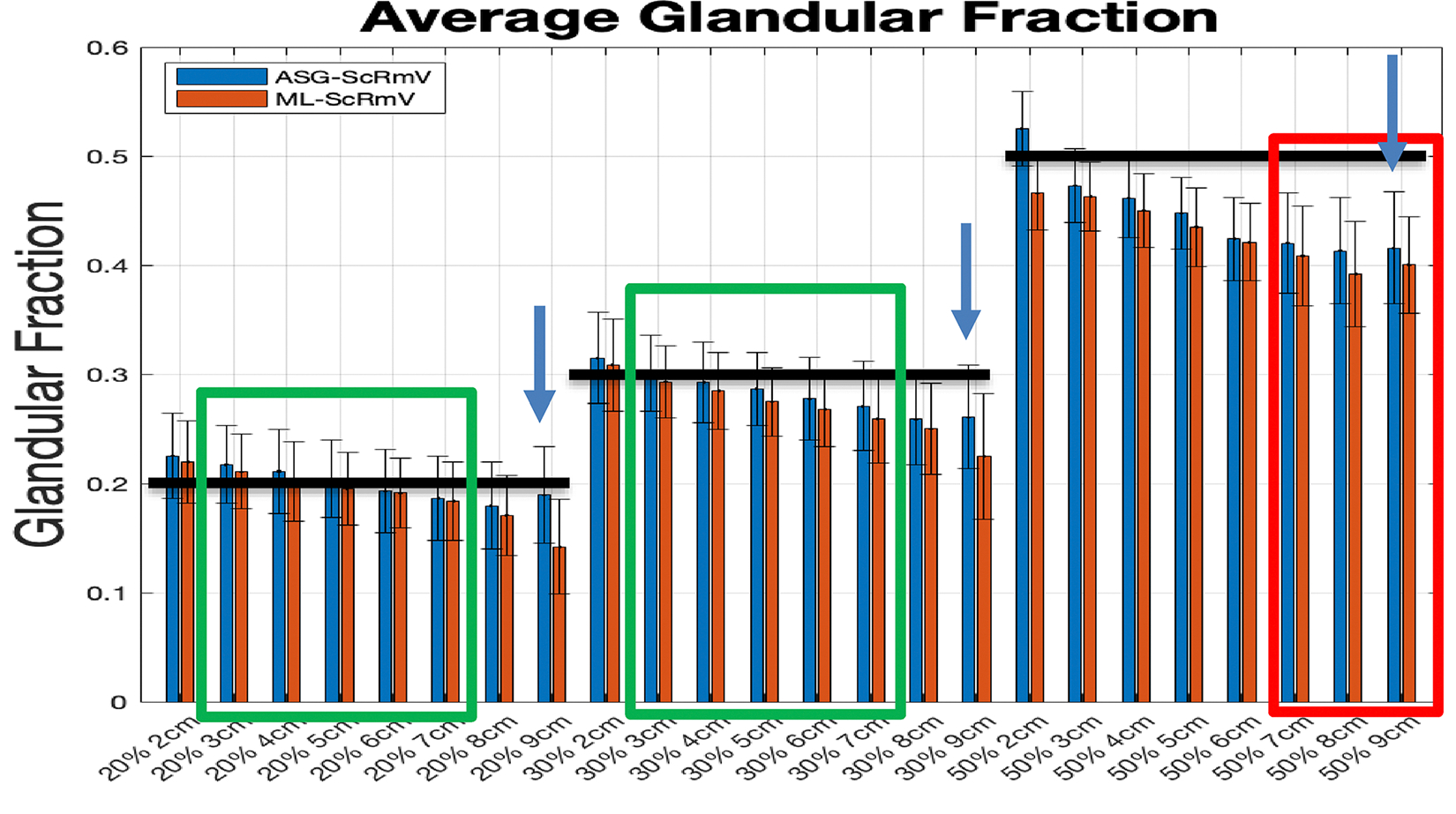
Average estimated Glandular Fraction with error-bars for *ML-ScRmv/ML-GF* which corresponds to software scatter removal followed by glandular fraction estimate *ASG-ScRmv/ML-GF* corresponds to anti-scatter grid removal technique followed by glandular fraction estimate. Black horizontal bars show the true value at each glandular fraction. The most likely range is 30% GF with 3–7 cm thickness (shown by the “most likely” green rectangles), [32, 33]. The thicker breasts 7–9 cm with 50% GF are highly unlikely cases (shown by the “Rare” red rectangle) [32, 33]. The algorithms perform well for 2–7 cm thickness for 20–30% GF with ASG-ScRmv slightly better for these cases. The algorithms show higher errors for thicker breast and higher GF, with ASG-ScRmv performing better than ML-ScRmv for the 8 cm and 9 cm cases. The W/Al spectrum is used

**Fig. 7:**
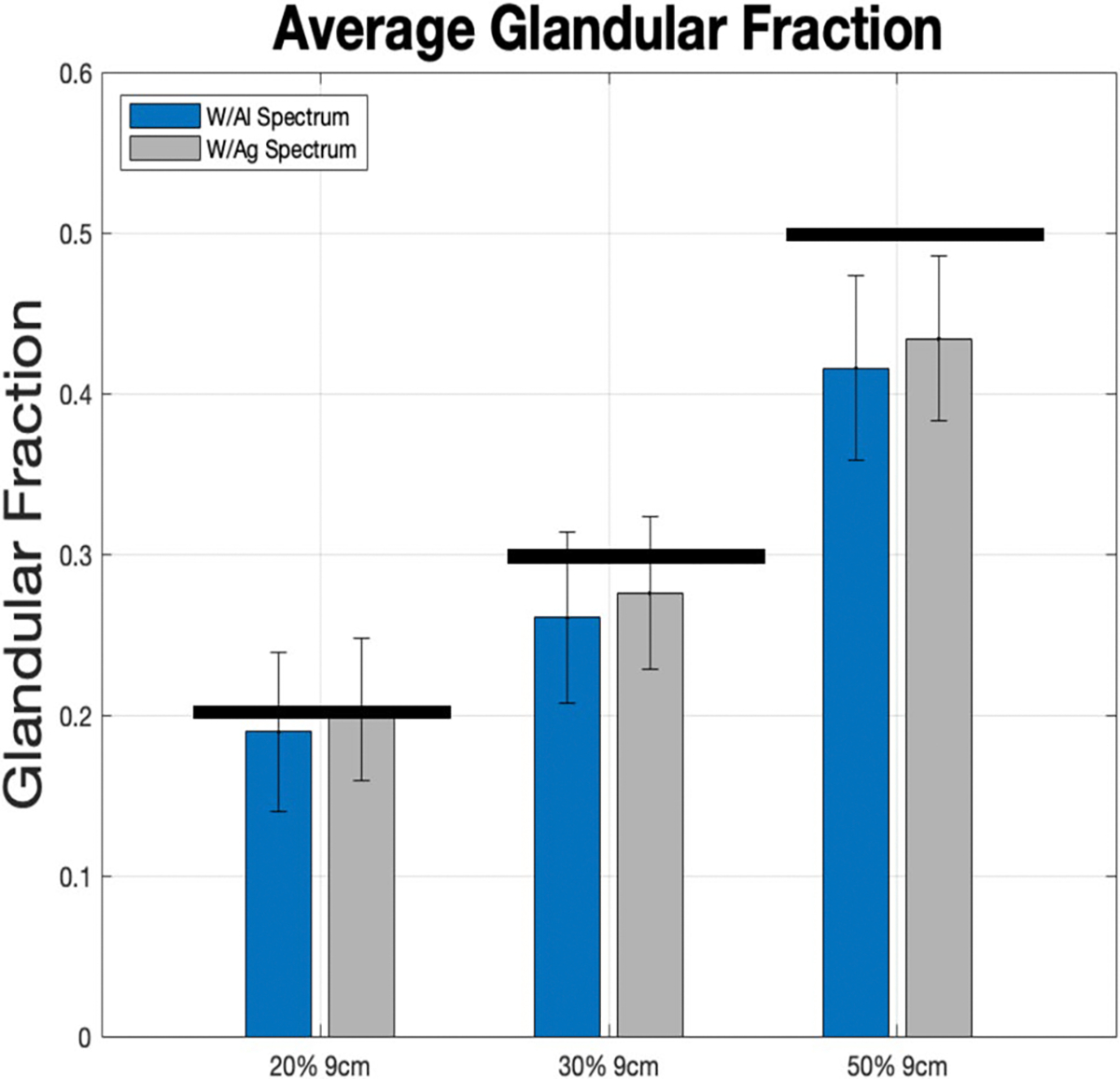
For the extreme 9 cm case, the *ASG-ScRmv/ML-GF* was repeated for a narrower W/Ag spectrum (gray bars). This shows better performance for W/Ag than with W/Al (blue bars)

Data for simulated phantoms with breast thicknesses of 2–9 cm was analyzed considering glandular fractions of 20%, 30%, and 50%. The results from the two algorithms, *ML-ScRmv/ML-GF and ASG-ScRmv/ML-GF*, using images from TOPAS Monte Carlo simulations, are shown in Figs. 6, 7. Figure 6 shows the average glandular fraction, standard deviation, and the true values (black bars) of 20%, 30%, and 50%. Note, the average values fall within one to two standard deviations of the true value of glandular fraction. For thickness range of 2–9 cm, the ML-ScRmv/ML-GF and ASG-ScRmv/ML-GF produced similar root mean square (RMSEs) of 7.6% and 6.6% deviations from the true GF value. The average absolute error were (*4.5* ± *2.3*)% and (*4.7* ± *2.8*)% respectively for the two algorithms. For the 9 cm (extreme) case, the error increased for all the glandular fractions, with the ASG showing better results than ML scatter removal. Errors were higher for an extreme case of 9 cm and the ASG-ScRmv/ML-GF performed better than ML-ScRmv/ML-GF. The 9 cm case was repeated with the W/Ag spectrum (as specified for the clinical case explored later in this work). As shown in Fig. 7, using a narrower X-ray spectrum reduced the error for the extreme 9 cm case. As shown with arrows in Fig. 6 and replotted (blue bars) in Fig. 7, with the ASG-ScRmv/ML-GF broader W/Al spectrum, the GF estimates were 19%,26% and 41.6% for actual GF of 20%, 30% and 50%. This indicates absolute errors of 5%, 13%, and 16% respectively for this 9 cm (extreme) case. With the narrower spectrum, the estimated values shown in gray bars in Fig. 7 improved to GF estimates of 20.3%, 27.6%, 43.4% for 20%, 30%, 50%, yielding absolute errors of 1.5%, 9%, and 13.2% respectively.

Figure 6 shows a decreasing trend in the estimated glandular fraction, increasing the error with increasing breast thickness for all glandular fractions. When a narrower X-ray spectrum was used the error was reduced. Increasing error with thicker breasts and broader spectrum suggests that beam hardening is a contributor to this systematic error. Beam hardening occurs as low-energy X-rays are preferentially absorbed as the X-ray propagates, resulting in a progressively higher average energy of the beam. Therefore the (energy dependent) attenuation coefficient for a given tissue type (whether glandular or adipose) can vary depending on their location on the path of the ray. In the current model, a single attenuation value (corresponding to the effective energy) was assumed for each tissue type. However, due to beam hardening, the actual attenuation will vary along the ray even within the same tissue, leading to errors in the glandular fraction estimation.

As expected, GF estimation yielded better results with ASG-based scatter removal (*ASG-ScRmv/ML-GF*, blue bars in Fig. 6) than with algorithmic scatter removal (*ML-ScRmv/ML-GF*, red bars), likely because the latter involves an additional estimation step. Remnant scatter remains a source of error for *ML-ScRmv/ML-GF*, particularly in thicker breasts.

**Fig. 8:**
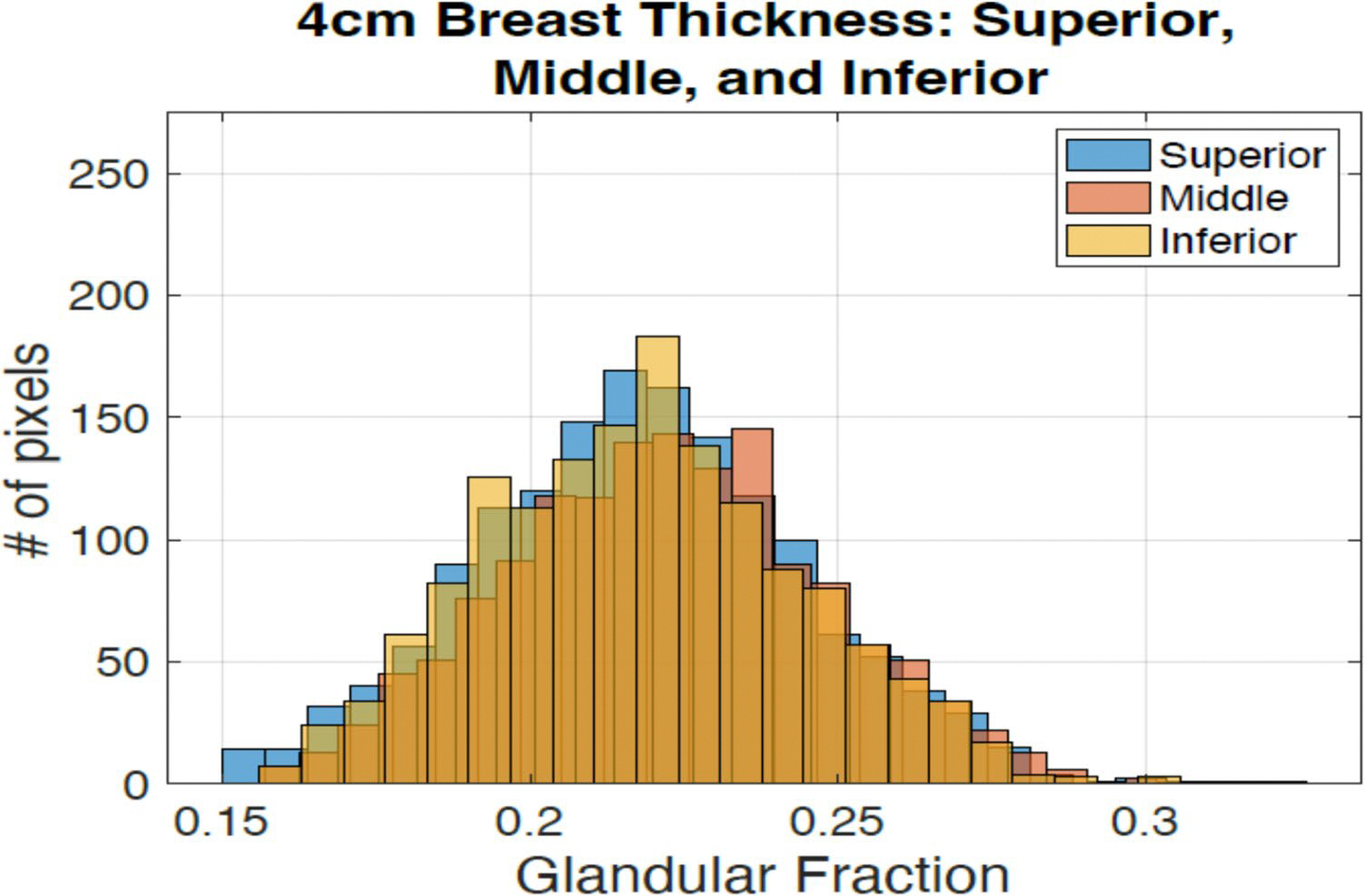
Histogram for different placement of a 20% GF case for a 4 cm thickness breast. The glandular tissue was placed 1 cm anterior to the center, at the center, and 1 cm posterior, away from the center

The Fig. 8 shows the histogram when the glandular tissue in a simulated phantom with compressed thickness of 4 cm with 20% glandular fraction is moved 1 cm closer to the surface, moved to the center and 1 cm away from the center. The average values are 0.215 ± 0.045, 0.217 ± 0.045, and 0.220 ± 0.045 for middle, superior, and inferior respectively. These results show a maximum difference of 2.3% in the average GF when varying glandular tissue location. These results show that there is no significant difference in glandular fraction estimation on z location of glandular tissue.

### Clinical images and microcalcification analysis

**Fig. 9:**
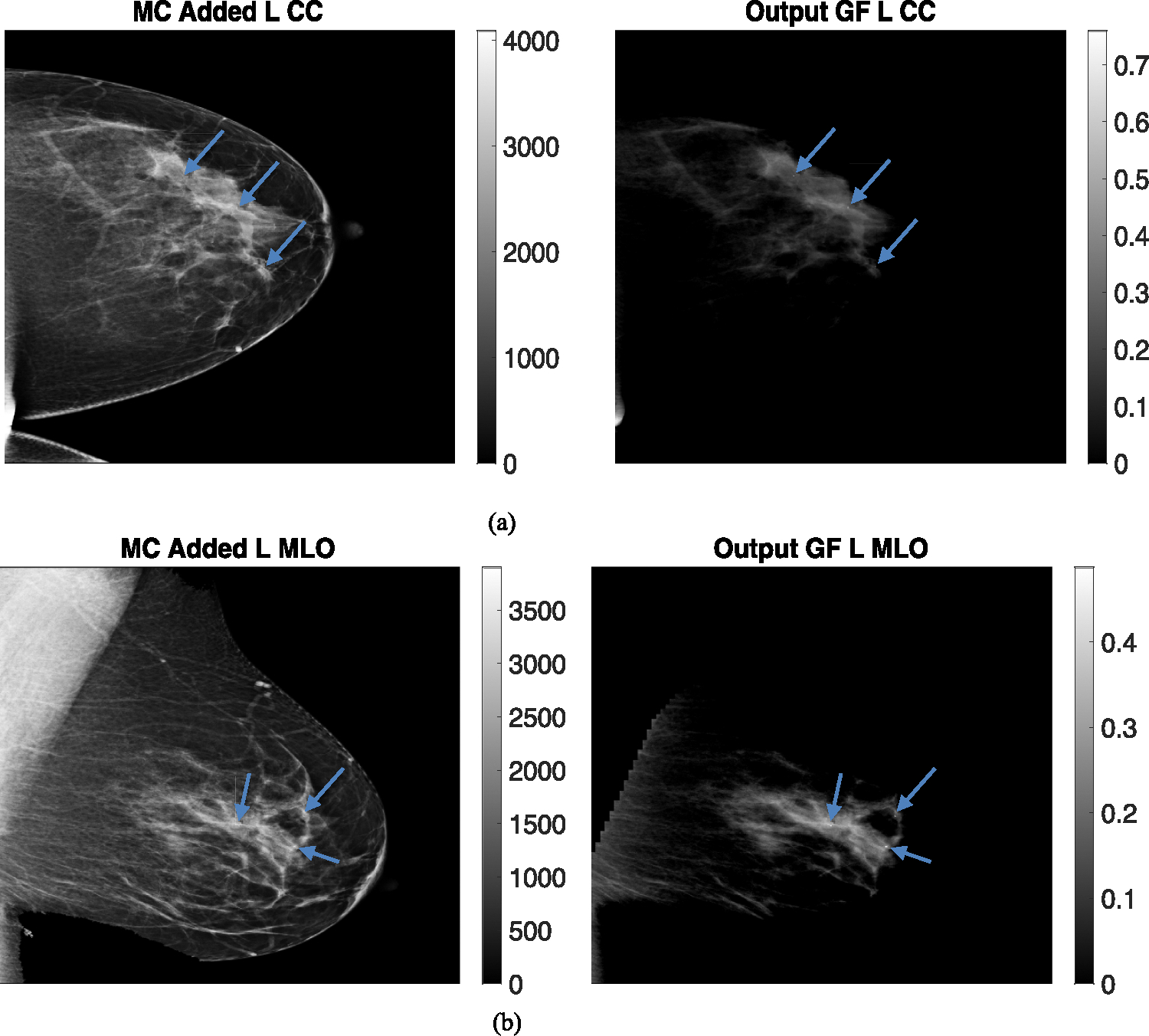
(Left) Microcalcification (MC) added clinical images and (Right) Output Glandular Fraction (GF) Images (output by algorithm). Different views are **a** left craniocaudal view, **b** left medio-lateral oblique view. Color bars in right images represent glandular fraction. Note the MCs that are virtually invisible in the clinical images become noticeable in GF-images

**Fig. 10:**
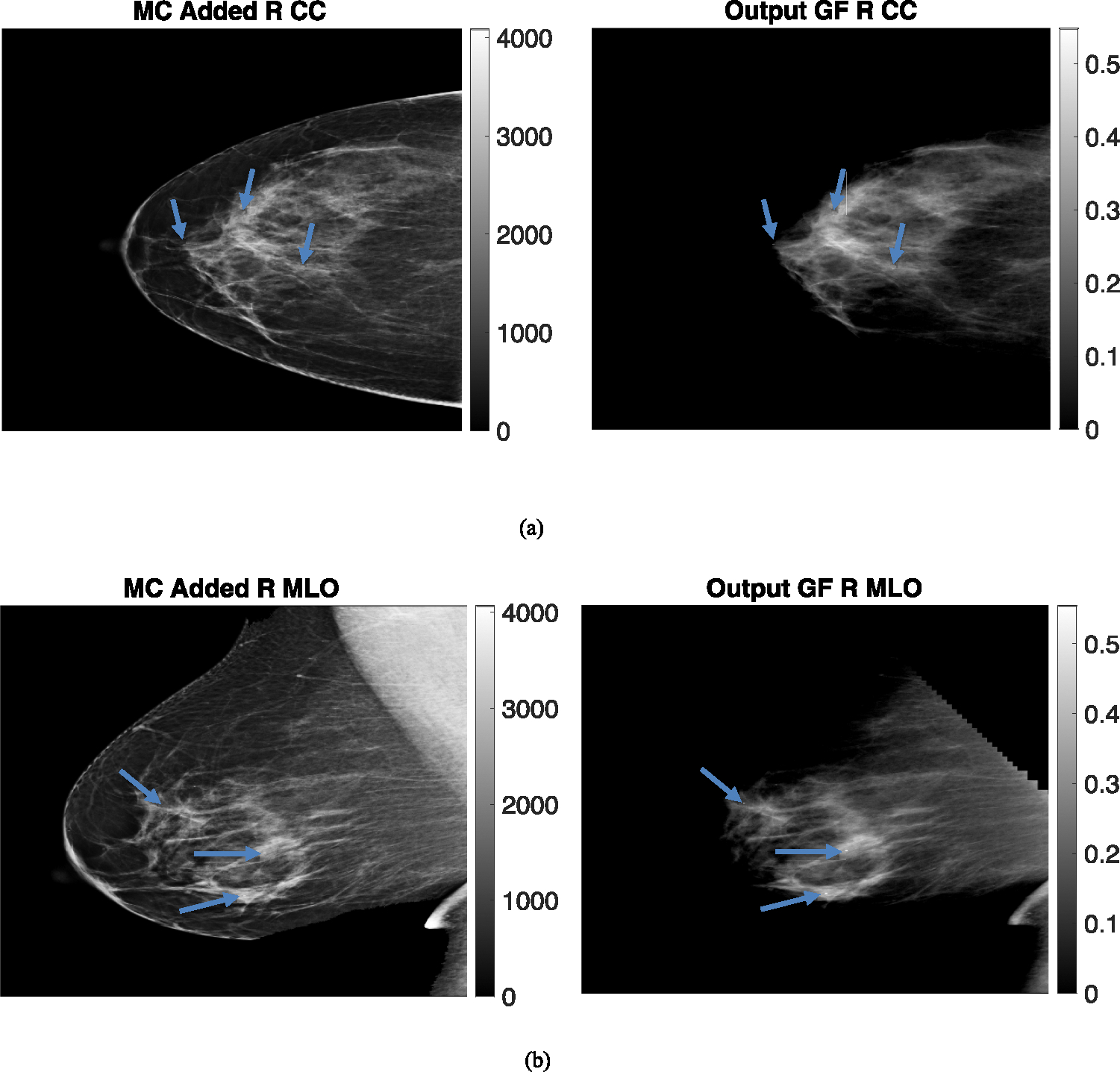
(Left) Microcalcification (MC) added clinical images and (Right) Output Glandular Fraction (GF) Images (output by algorithm). Different views **a** right craniocaudal view of 9 cm breast and **b** right mediolateral oblique view. Color bars in right images represent glandular fraction. Note the MCs that are virtually invisible in the clinical images become noticeable in GF-images

The breast thicknesses varied between 86 and 98 mm, for the clinical datasets, depending on the view, and the peak energies of the spectrums were 33 kVp, 34 kVp, 35 kVp, and 34 kVp for R CC, L MLO, L CC, and R MLO respectively. Figures 9 and 10 display clinical images with added microcalcifications (following steps 1–3 outlined in the methods) and the corresponding output glandular fraction (GF) images generated by the ML-GF algorithm for all four views (the right column).

In these images, the GF varies from 40% in the brightest regions to around 10% towards the chest wall. Since the true glandular fraction values are unknown, the error cannot be estimated. However, the average of around 30% in the CC view aligns with the average glandular fraction range found in the literature [3].

**Fig. 11:**
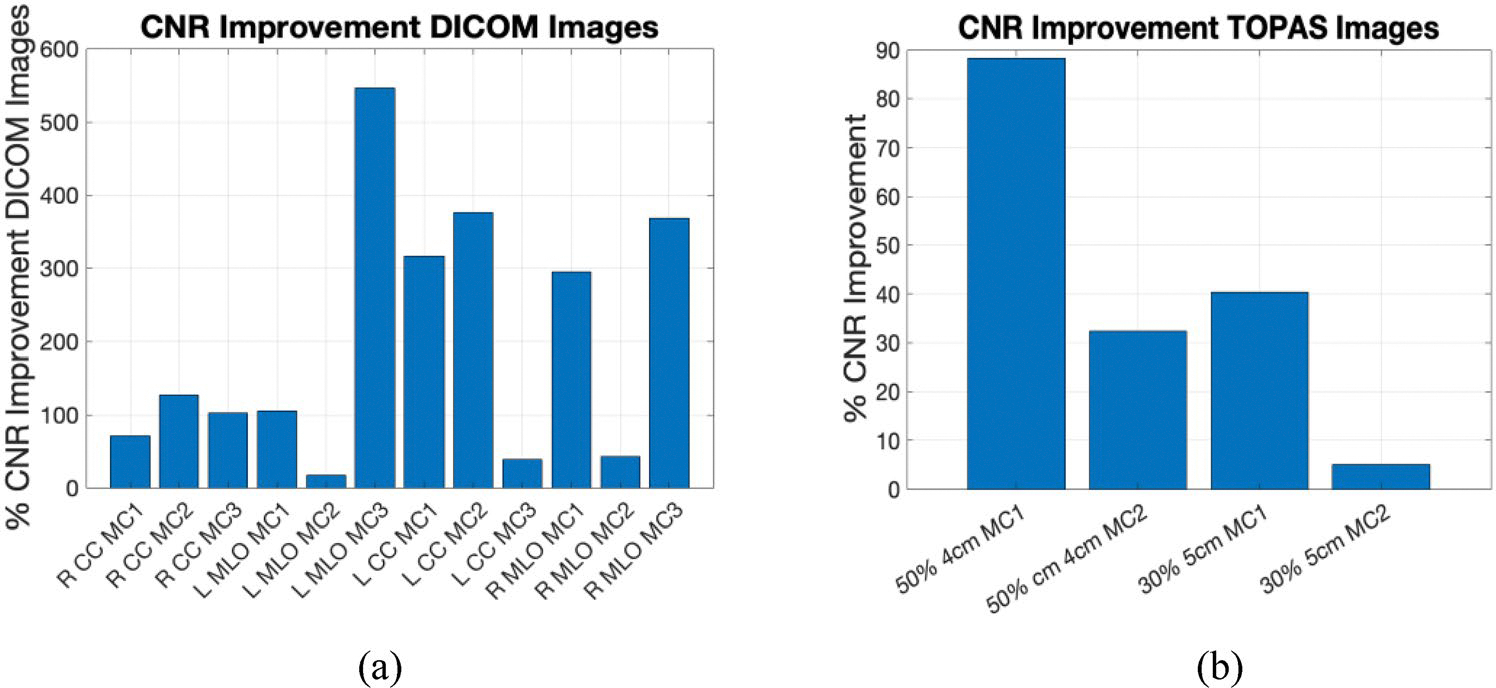
Percent improvement on contrast-to-noise ratio for Microcalcifications **a** on GF estimated images from clinical images **b** on the GF-images outcomes for TOPAS images

The GF images for calcifications in Figs. 9, 10 show some visibility improvement over the displayed clinical images. Note no special processing was applied on the GF images. While visibility is still subtle because the macrocalcifications were very small, (1 mm diameter) there were significant CNR improvements as shown in Fig. 11. Figure 11a shows the contrast-to-noise ratio on all the three microcalcifications that were added in each image, for a total of 12 separate calcifications across the images. The CNR improvement was nearly 550% in the L MLO (mediolateral oblique view) while the lowest improvement was a 17.5% improvement in one of the microcalcifications in the same L MLO view. Figure 11b also show improvement in contrast-to-noise for microcalcifications for simulated digital breast phantoms for 50% GF and 30% GF images. These were computed with respect to unprocessed (raw count) images.

## Discussion

This work demonstrates that a comprehensive physics-based model can accurately estimate glandular fraction (GF) in most cases. Two GF estimation approaches were evaluated: one using ASG-based scatter removal and the other using algorithmic scatter removal. Both methods yielded promising results, with average absolute errors of 4.5% and 4.7%, and RMSE values of 6.6% and 7.6%, respectively across simulated cases with simulated compressed breast phantom thicknesses from 2 to 9 cm using a broad W/Al spectrum. These results outperform the 13.6% error reported by Heine et al. [10] on clinical data, though it is be noted that our evaluation was conducted on simulated data.

For the simulations with a range of breast thickness of 2–9 cm, a wider spectrum with W/Al was used to test the effective energy method of incorporating the effect of the polychromatic beam. For 9 cm, both the W/Al and W/Ag spectrum were tested. For the clinical cases the W/Ag was used, as specified by the DICOM header for the clinical datasets.

As detailed in the Results section, our model assumes a single-attenuation value (at the effective-energy) for each of the tissue type. However, due to beam hardening, the actual attenuation will vary along the ray even within the same tissue type (whether adipose or glandular fraction), leading to errors in the glandular fraction estimation.

The *ML-ScRmv* scatter removal method will be utilized to estimate the glandular fraction (GF) in mammographic acquisitions when an anti-scatter grid is not used to reduce the dose. However, GF estimation performed better with ASG-based scatter removal (*ASG-ScRmv/ML-GF*, blue bars in Fig. 6) than with algorithmic removal (*ML-ScRmv/ML-GF*, red bars), because the latter involves an additional estimation step and remaining scatter increases the estimation error, especially for thicker breasts.

The methods here show there is the necessity of a comprehensive model to achieve a quantitatively accurate GF. However, one of the limitations of this work is the $I_{0}$ that is needed in the estimation of the GF, as well as access to raw data. This requirement limited the clinical data analysis to a single case with publicly accessible raw linear images. The $I_{0}$ was estimated from the background in this case. The availability of four different views of the same patient led to cases of different overlaps of tissue and created four different unique “clinical cases” of pixelwise GF to evaluate.

The focal spot blur is not explicitly simulated but its effect is minimal since the object is next to the detector, making the magnification small. For example, for a typical source-detector distance 70 cm, the worst case (for top surface of breast) effect of the focal spot blur of 300 μm on the detector for a simulated breast phantom with thickness 4 cm with a 1.5 cm gap to the detector will be 5.5/(70–5.5) × 300 μm = 25.6 μm, which is subpixel for a 50 μm flat panel detector. Smaller detector thickness also will not have a significant effect on the GF estimation in terms of detection sensitivity compared to what was shown in the simulations, because the simulated count levels (125 million × 8 energy bins) are several orders lower than true clinical count levels.

Although other robust and fast quadratic optimizers, such as Levenberg-Marquard exist, the use of Newton’s method was inspired by similar methods used in 3D MLEM reconstruction methods [49]. The algorithm consistently converged rapidly within seconds in all cases demonstrating robustness.

The slower of the two methods, the *ML-ScRmv/ML-GF* code (in Matlab) converged within 4 s for the simulated cases and 15 s for the clinical cases on a MacBook Air, M1.

The count levels for clinical cases are significantly higher than simulated. Therefore, it is believed that the method will be robust to system-to-system differences such as source-detector distance, target/filter combinations and maintain similar margins of accuracy for other systems.

Qualitatively, the pixel-wise GF corresponded to excellent segmentation of glandular tissue versus background (Figs. 8 and 9). The GF values obtained were well within the expected values clinically. Some data required by the complete algorithm maybe absent such as the paddle thickness. In such cases, one can attempt to find if the specific information is available for the system described in the DICOM header. If that fails, one may assume standard values in the literature for the system. Errors in paddle thickness adds only a small scaling error in the estimates for smaller breast thickness. Finally, the ML-GF images seem to make visible realistic small micro-calcifications (which were virtually invisible in displayed clinical images) with the CNR improved on average 200%. This makes it a potentially viable image to show physicians in addition to the high contrast clinical images.

The methods can be directly applied to other advanced imagers, such as attenuation image for X-ray interferometry system.

One of the limitations of the work is that the input spectrum was not varied according to thickness and glandularity for the simulations. The AEC was also not modeled.

Another limitation relates to marginal pixels. In this study, semi-cylindrical objects with centrally located glandular tissue were used (Fig. 2), so the algorithm did not show the edge effects or errors due to thinning of breast. But for a compressed breast, at the edges the tissue curves out and X-rays pass through air gaps, especially around the nipple. This results in an error in the glandular fraction estimation for those pixels, as the thickness measurement includes these air gaps (beyond ray B in Fig. 1). The size of this marginal region depends on the breast’s length, thickness, and shape after compression.

In the future, the Graff mathematical phantom [50] and FEM package, FEBio [51], will be used to simulate compressed breasts of various sizes to investigate GF estimation at the marginal pixels. One strategy to address the thinning issue involves identifying the marginal pixels and applying tissue equalization methods—commonly used for display purposes [26] and volume glandular fraction estimates [9, 27]—before calculating the GF estimates. Another approach to consider is incorporating alternative thickness and attenuation estimates into the ML-GF model by using our thickness estimation algorithm [29] and apply this dual estimation method specifically at the marginal pixels. Estimation for the central pixels will continue to follow the approach outlined here.

## Conclusion

A ML algorithm was developed to estimate the pixel wise glandular fraction of an object. The ML software was evaluated with images generated from TOPAS Monte Carlo simulations and the estimated glandular fraction converges to the true glandular fraction with an RMSE of 6.6% and 7.6% for anti-scatter-grid and software-based method respectively for a range of 2–9 cm compressed breast thicknesses. Average absolute errors were 4.5% and 4.7% respectively. Furthermore, this maximum-likelihood estimation method showed an average of 200% improvement in contrast-to-noise on microcalcification for the clinical images. The realistic micro-calcifications were nearly invisible in original displayed imaged but were visible in the glandular fraction images. This is further important because there has been shown to be a proportional relationship between glandular fraction and microcalcifications and breast cancer risk.

## Figures and Tables

**Table 1:** Percent errors between total counts lost (as defined in Eq. 9) due to absorption and scatter with poly-energetic spectrum vs effective energy approximation) for different glandular fraction (GF) and compressed breast thickness (T)

T	2 cm (%)	3 cm(%)	4 cm (%)	5 cm (%)	6 cm (%)	7 cm (%)	8 cm (%)	9 cm (%)

GF								
20%	0.83	0.90	0.83	0.76	0.70	0.63	0.57	0.59
30%	0.78	0.85	0.83	0.75	0.69	0.61	0.55	0.57
50%	0.68	0.87	0.83	0.75	0.67	0.61	0.52	0.56
